# Wallenberg Syndrome Secondary to Vertebrobasilar Aneurysm Associated With Subclavian Steal Syndrome

**DOI:** 10.7759/cureus.72623

**Published:** 2024-10-29

**Authors:** Francisco Castañeda Aguayo, Andrés Jaime Aguirre, Isai Garcia, Gervith Reyes Soto, Carlos Catillo-Rangel, Carlos Castillo Soriano, Nasser M F El-Ghandour, Matias Baldoncini, Andreina Rosario Rosario, Manuel de Jesus Encarnacion Ramirez

**Affiliations:** 1 Department of Neurosurgery, Centro Medico Nacional 20 de Noviembre, Instituto de Seguridad y Servicios Sociales de los Trabajadores del Estado (ISSSTE), Mexico City, MEX; 2 Department of Neurosurgery, Centro Médico Nacional 20 de Noviembre, Instituto de Seguridad y Servicios Sociales de los Trabajadores del Estado (ISSSTE), Mexico City, MEX; 3 Department of Head and Neck, Unidad de Neurociencias, Instituto Nacional de Cancerología, Mexico City, MEX; 4 Department of Neurosurgery, Servicio of the 1ro de Octubre Hospital, Instituto de Seguridad y Servicios Sociales de los Trabajadores del Estado (ISSSTE), Mexico City, MEX; 5 Department of Neurosurgery, Tecnológico de Monterrey Campus Estado de México, Mexico City, MEX; 6 Department of Neurosurgery, Faculty of Medicine, Cairo University, Cairo, EGY; 7 Laboratory of Microsurgical Neuroanatomy, School of Medicine, University of Buenos Aires, Buenos Aires, ARG; 8 Medical School, Autonomous University of Santo Domingo (UASD), Santo Domingo, DOM; 9 Department of Neuroscience, Instituto Nacional de Cancerología, Mexico City, MEX; 10 Digital Anatomy, United Nations Educational, Scientific and Cultural Organization, Paris, FRA; 11 Neurological Surgery, Peoples' Friendship University of Russia, Moscow, RUS

**Keywords:** subclavian steal syndrome, vertebral artery, vertebral artery calcification, vertebrobasilar aneurysm, wallenberg syndrome

## Abstract

Wallenberg syndrome, also known as lateral medullary syndrome, is a rare condition affecting the vertebrobasilar circulation, causing symptoms such as vertigo, nystagmus, dysarthria, and hemifacial weakness. Typically linked to ischemic strokes, it can also arise from vertebrobasilar aneurysms. In rare cases, subclavian steal syndrome (SSS), involving retrograde flow in the vertebral artery due to subclavian stenosis, complicates the picture, as observed in this case of a 66-year-old woman with both conditions and a vertebrobasilar aneurysm. This study was conducted at the Neurosurgery Department of Centro Médico Nacional 20 de Noviembre, Mexico City. The patient, a 66-year-old woman with hypertension and chronic smoking, presented with vertigo, diplopia, and quadriparesis. Imaging revealed a vertebrobasilar aneurysm and SSS. Despite recommendations for further invasive studies, the patient declined angiography and therapeutic interventions, opting for voluntary discharge without treatment. This case underscores the rare association of Wallenberg syndrome with a vertebrobasilar aneurysm and SSS. Hemodynamic stress from retrograde vertebral artery flow likely contributed to aneurysm formation. Advanced imaging is vital for diagnosis, and while the patient refused treatment, multidisciplinary management, including future innovations such as three-dimensional printing and endovascular techniques, holds promise for improving outcomes in such complex cases.

## Introduction

Wallenberg syndrome, also known as lateral medullary syndrome, is a rare condition that primarily affects the vertebrobasilar circulation. It is characterized by a clinical presentation that includes vertigo, nystagmus, dysarthria, dysphagia, and hemifacial weakness, among other symptoms. Although it is generally associated with an ischemic stroke in the vertebral or cerebellar artery, there are other less common causes, such as compression from aneurysms in the vertebrobasilar region or arterial dissections [1]. In this context, the coexistence of a vertebrobasilar aneurysm with subclavian steal syndrome (SSS) adds an unusual element to the clinical presentation, as observed in the case we report.

The vertebrobasilar system supplies blood to important structures of the central nervous system, such as the brainstem, cerebellum, and posterior portions of the brain. The vertebral arteries, which originate from the subclavian artery, traverse the neck and join at the base of the skull to form the basilar artery. This anatomical structure, although robust, is susceptible to a variety of hemodynamic and structural alterations, such as aneurysms, dissections, and arteriovenous malformations [2]. Vertebrobasilar circulation aneurysms represent only 0.5% of all intracranial aneurysms, making them a rare but highly relevant entity due to the challenges of surgical management and the high mortality risk they present [3]. Even more uncommon is the formation of aneurysms as a result of SSS, a condition in which stenosis or occlusion of the subclavian artery causes retrograde flow in the ipsilateral vertebral artery [4].

SSS is a phenomenon that generally affects patients with advanced atherosclerotic disease or congenital anomalies of the subclavian artery. In this syndrome, occlusion or stenosis of the proximal subclavian artery causes retrograde flow in the ipsilateral vertebral artery, resulting in a “steal” phenomenon of blood from the posterior circulation. Although most patients with this syndrome remain asymptomatic, some develop neurological symptoms as a result of vertebrobasilar ischemia, manifesting as vertigo, dysarthria, diplopia, or weakness [5]. In rare cases, as presented in this report, this hemodynamic phenomenon can contribute to the formation of aneurysms at the vertebrobasilar junction, further complicating the clinical scenario and therapeutic management.

This case involves a 66-year-old woman with a history of hypertension and chronic smoking, who presented with a clinical picture of vertigo, double vision, postural instability, and disproportionate quadriparesis, leading to the diagnosis of a vertebrobasilar aneurysm. What distinguishes this case is the coexistence of SSS, a condition in which the left vertebral artery was completely occluded before entering the C2 transverse foramen, likely due to significant calcification of the left subclavian artery. This unusual hemodynamic finding was identified through imaging studies, including magnetic resonance angiography (MRA) and three-dimensional (3D) reconstructions, which confirmed the absence of flow in the left vertebral artery and the presence of a partially thrombosed aneurysm at the vertebrobasilar junction [6].

Aneurysm formation at the vertebrobasilar junction is rare, and its association with SSS has been documented in only a small number of cases in the literature [7]. It has been proposed that retrograde flow in the vertebral artery, caused by subclavian stenosis, increases hemodynamic stress at the vertebrobasilar junction, contributing to aneurysm formation in this region [8]. The treatment of these aneurysms is complex due to their deep anatomical location and proximity to critical structures, increasing the risk of intraoperative complications. Although endovascular techniques have significantly improved the prognosis of vertebrobasilar aneurysms, surgical management remains challenging in many cases, particularly when complicated hemodynamic factors such as SSS are present [9].

This case highlights the importance of considering SSS in patients presenting with vertebrobasilar aneurysms, especially when significant hemodynamic alterations are found in the vertebral circulation.

## Case presentation

Study setting

This study was conducted at the Neurosurgery Department of Centro Médico Nacional 20 de Noviembre, Instituto de Seguridad y Servicios Sociales de los Trabajadores del Estado (ISSSTE), Mexico City, a tertiary care institution equipped with advanced diagnostic tools. The patient received thorough diagnostic evaluation and clinical management during her stay in the neurosurgical unit.

Patient

The patient was a 66-year-old woman with a history of long-standing hypertension and chronic smoking, both of which are known risk factors for vascular disease. She presented with a progressive neurological deficit, characterized by vertigo, diplopia, and weakness in all four limbs over eight months. This case was selected for reporting due to its rarity and the complex interplay between the vertebrobasilar aneurysm and the SS, making it an unusual and educational case for the scientific community.

Clinical assessment

Upon admission, the patient underwent a detailed neurological examination, which revealed signs consistent with posterior circulation involvement. The Glasgow Coma Scale (GCS) score was 14 points (indicative of mild disorientation). The Romberg test was positive, demonstrating postural instability. The patient exhibited lateralization of gait to the right, consistent with vestibular dysfunction. On motor examination, right hemibody strength was 4/5, while left hemibody strength was 3+/5, indicating quadriparesis was more severe on the left side. Sensory examination was not accurately assessed due to poor cooperation from the patient.

Diagnostic imaging

**Figure 1:**
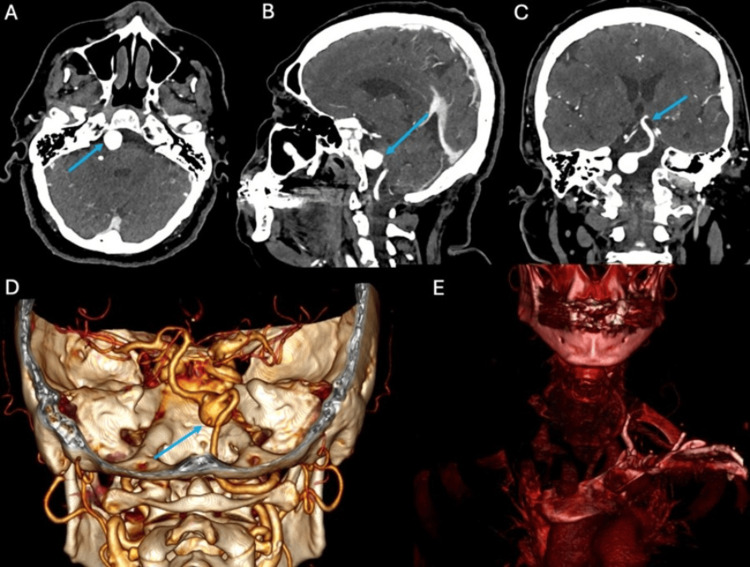
Cerebral angiography in axial (A), sagittal (B), and coronal (C) views, as well as three-dimensional reconstruction, showing a right vertebrobasilar aneurysm, absence of flow in the left vertebral artery (D), and calcification of the left subclavian trunk (E).

Imaging studies were crucial in identifying the underlying pathology. Initial CT imaging identified a saccular lesion located at the right vertebrobasilar junction, measuring approximately 1.4 × 1.3 × 1.3 cm, suggestive of a vertebrobasilar aneurysm (Figures 1A-1C). 3D reconstructions revealed a calcified lesion at the left clavicular level, compromising the flow in both the left subclavian artery and the vertebral artery (Figure 1D). This finding indicated the presence of SSS, further supporting the hypothesis of hemodynamic alterations contributing to aneurysm formation (Figure 1E).

**Figure 2:**
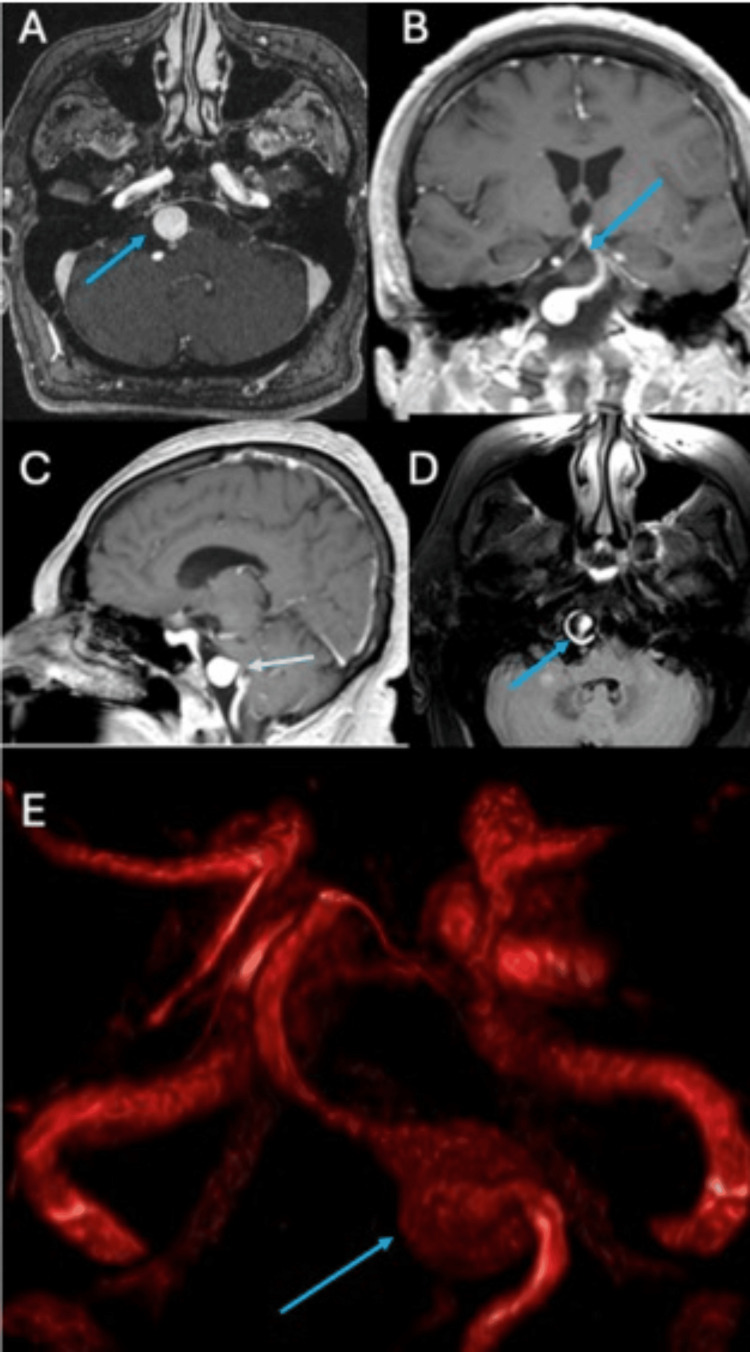
Magnetic resonance images with T1 gadolinium-weighted sequences in axial (A), coronal (B), and sagittal (C) views, along with fluid-attenuated inversion recovery-weighted axial section (D), showing enhancement at the vertebrobasilar level corresponding to an aneurysm. Three-dimensional reconstruction showing the anterior and posterior cerebral circulation, with the presence of a right vertebrobasilar aneurysm and an absence of flow in the left vertebral artery (E).

MRA was performed to confirm the presence of the vertebrobasilar aneurysm and assess the flow in the vertebral arteries. The imaging confirmed a partially thrombosed aneurysm and an absence of flow in the left vertebral artery, suggesting occlusion at the subclavian level (Figure 2).

**Figure 3:**
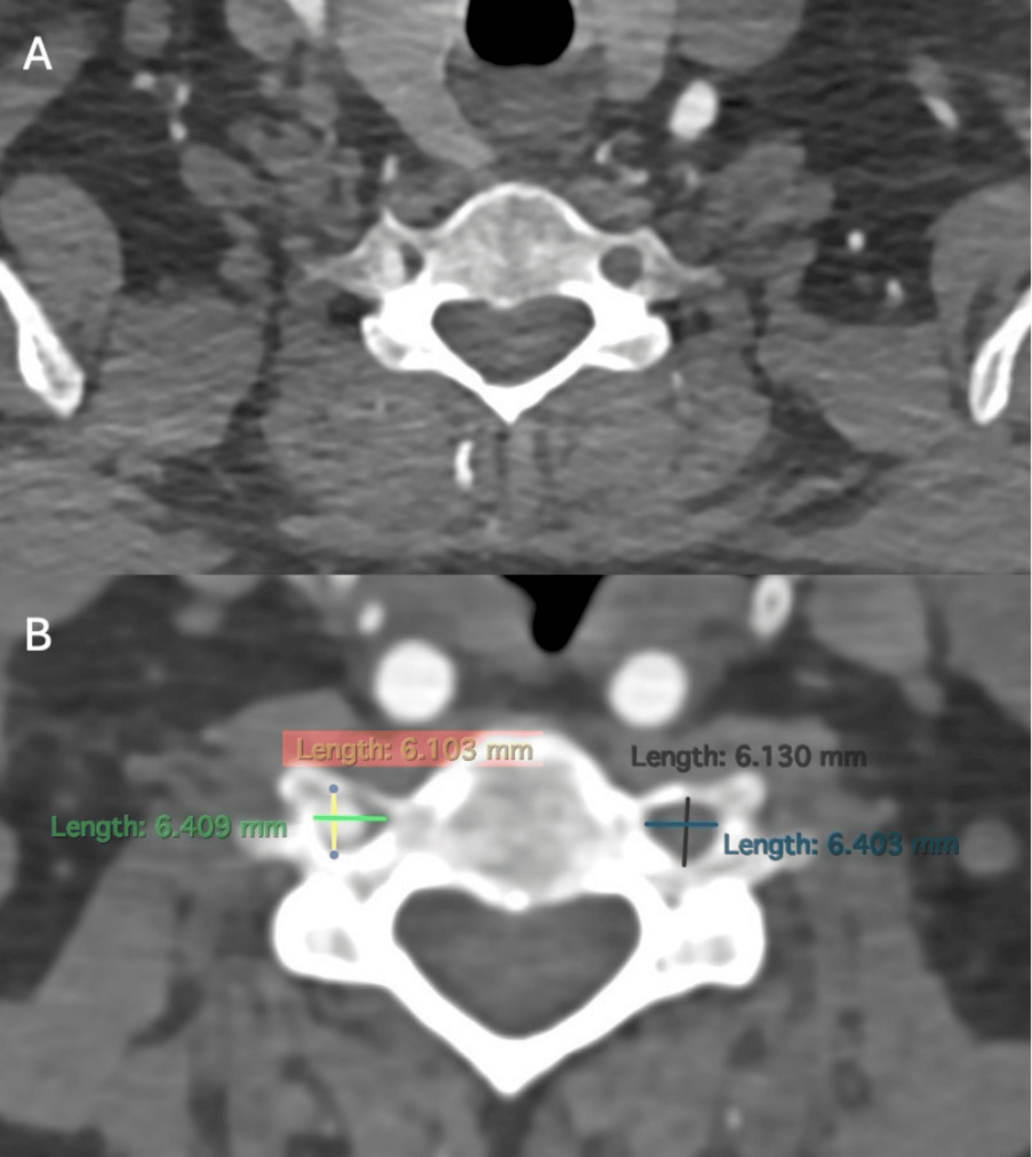
Axial section of cervical angiography showing contrast presence in the right vertebral artery and absence on the left side (A). Comparison of measurements of the transverse foramina, with no significant differences between both sides (B).

Bilateral transverse foramina were compared using high-resolution imaging, and no significant anatomical differences were found between sides. This ruled out structural narrowing as a contributing factor (Figure 3).

Hemodynamic evaluation

Hemodynamic analysis played a pivotal role in understanding the development of the vertebrobasilar aneurysm. The absence of flow in the left vertebral artery was considered secondary to the calcification and occlusion of the subclavian artery. The retrograde flow resulting from SSS likely caused increased hemodynamic stress at the vertebrobasilar junction, which is proposed as the origin of the aneurysm. Doppler ultrasound studies and MRA confirmed the absence of anterograde flow in the left vertebral artery, with evidence of compensatory increased flow in the right vertebral artery. Doppler studies demonstrated a significant pressure gradient between the upper limbs, with a >10 mmHg difference, a classic finding in SSS.

Proposed interventions

Given the patient’s complex vascular anatomy and the high risk associated with vertebrobasilar aneurysms, a cerebral angiography was proposed to further characterize the aneurysm and assess possible therapeutic options. However, the patient and her family declined invasive diagnostic procedures and opted for voluntary discharge from the hospital, thus precluding surgical or endovascular intervention.

Ethical considerations

The patient’s consent was obtained for the publication of this case report. All identifying information was anonymized, and the case report adhered to the ethical guidelines for medical research involving human subjects, as outlined by the Declaration of Helsinki. The voluntary refusal of further invasive diagnostic and therapeutic procedures was respected, and the patient’s decision was documented in her medical records.

## Discussion

The case presented here is a valuable contribution to the limited body of literature concerning vertebrobasilar aneurysms secondary to SSS, an extremely rare phenomenon with significant clinical and surgical implications. The complexity of this case lies in the unusual combination of two rare vascular conditions that complicate both the clinical presentation and the treatment options [10].

Pathophysiology

The vertebrobasilar system plays a critical role in providing blood flow to vital structures of the brainstem, cerebellum, and posterior cerebral cortex. Any disruption of flow in this system can result in serious neurological deficits [11,12]. The pathophysiology behind the development of vertebrobasilar aneurysms, particularly those associated with SSS, involves abnormal hemodynamic stresses placed on the arterial walls [8,9].

SSS occurs when stenosis or occlusion of the subclavian artery, typically due to atherosclerosis or less commonly congenital abnormalities, leads to retrograde flow in the vertebral artery on the ipsilateral side [9]. This phenomenon is exacerbated when the subclavian artery has significant stenosis, and the vertebral artery is forced to compensate for the reduced perfusion to the arm by drawing blood away from the posterior circulation [11,13]. This retrograde flow increases the hemodynamic stress at the junction where the vertebral arteries join to form the basilar artery, potentially predisposing the patient to aneurysm formation [6].

In this case, the retrograde flow through the left vertebral artery due to the subclavian steal phenomenon likely contributed to the formation of a saccular aneurysm at the vertebrobasilar junction. The abnormal flow dynamics resulting from the subclavian stenosis increased the likelihood of aneurysm development, as confirmed by the absence of anterograde flow in the left vertebral artery on imaging [12-14]. This mechanism is consistent with the pathophysiology described in the literature, where hemodynamic stress is considered a key factor in aneurysm formation in this location [15,16].

The clinical presentation of vertebrobasilar aneurysms can be varied, depending on the size, location, and associated hemodynamic changes. In this case, the patient presented with symptoms of Wallenberg syndrome, which include vertigo, diplopia, and quadriparesis. Wallenberg syndrome, or lateral medullary syndrome, is typically associated with infarcts in the posterior circulation, particularly the vertebral artery or posterior inferior cerebellar artery [3,14,17].

However, in rare cases, unruptured aneurysms in the vertebrobasilar region can present similarly to ischemic events due to compression of brainstem structures or reduced perfusion from hemodynamic disturbances. In this case, the combination of vertebrobasilar aneurysm and SSS led to a complex presentation that mimicked ischemic events in the posterior circulation [18,19]. The lack of ruptured aneurysm signs, such as subarachnoid hemorrhage, delayed the diagnosis and complicated the clinical evaluation [9].

The patient’s history of hypertension and smoking are known risk factors for vascular diseases, including aneurysm formation and atherosclerosis, further contributing to her susceptibility to both conditions. The chronicity of her symptoms, lasting for eight months, suggests that the aneurysm and subclavian stenosis had been progressing slowly, allowing time for the development of compensatory mechanisms such as retrograde flow in the vertebral artery [15,20].

Imaging played a critical role in the diagnosis and understanding of this patient’s condition. Initial cranial CT revealed a saccular lesion at the vertebrobasilar junction, which was later confirmed to be a vertebrobasilar aneurysm. The MRA further clarified the nature of the lesion, showing partial thrombosis and absence of flow in the left vertebral artery due to occlusion from subclavian stenosis [16,17,21].

The use of 3D reconstruction imaging provided additional insights into the anatomical abnormalities contributing to the patient’s condition. The calcified lesion at the left subclavian artery level was identified as the source of the subclavian steal phenomenon, and its impact on vertebral artery flow was confirmed [22]. The absence of significant anatomical differences in the transverse foramina ruled out structural abnormalities that could have otherwise contributed to vertebral artery hypoplasia or other flow restrictions [23].

The identification of SSS was confirmed by Doppler ultrasound, which showed a significant pressure gradient between the arms, a hallmark finding in patients with this condition. The hemodynamic changes observed in this patient, particularly the retrograde flow in the vertebral artery, are well-documented in the pathophysiology of SSS and are directly linked to the aneurysm formation [3,24,25].

The treatment of vertebrobasilar aneurysms associated with SSS is challenging, particularly because both conditions pose distinct risks and require different approaches. The primary goal in treating vertebrobasilar aneurysms is to prevent rupture, which carries a high mortality rate due to the critical location of these aneurysms near the brainstem [10]. However, addressing the hemodynamic changes caused by SSS is also crucial to reducing the ongoing stress on the vertebral and basilar arteries [26].

Endovascular treatment, such as coiling or stenting, is typically the first-line option for vertebrobasilar aneurysms, as it is less invasive and carries a lower risk of complications compared to open microsurgery. In cases where SSS contributes to aneurysm formation, angioplasty or stenting of the subclavian artery may be required to restore normal flow and reduce the retrograde pressure in the vertebral artery. However, endovascular techniques are not without risks, and the potential for thromboembolism or vessel rupture must be carefully considered [27,28].

Open microsurgery is reserved for cases where endovascular treatment is not feasible, either due to the anatomy of the aneurysm or other factors. Microsurgical clipping of the aneurysm, along with possible bypass surgery to restore vertebral artery flow, offers a definitive solution but carries significant risks, particularly in the vertebrobasilar region. The deep location of the aneurysm, proximity to critical structures, and potential for brainstem ischemia make this a high-risk procedure that requires careful patient selection [19,29,30].

In this case, the patient’s refusal of further invasive procedures limited the ability to pursue definitive treatment options. While conservative management was the only option remaining, the long-term prognosis remains uncertain, and the risk of aneurysm rupture persists.

Limitations

The study is based on a single case, which limits the generalizability of the findings. While the rarity of this condition justifies the focus on an individual case, broader studies involving multiple cases are necessary to confirm the clinical and pathophysiological correlations proposed.

The refusal of the patient to undergo further invasive diagnostic procedures, such as angiography, and therapeutic interventions, such as endovascular or surgical treatments, limits the ability to fully assess the aneurysm’s hemodynamic characteristics and therapeutic outcomes. This restricted the depth of the study in evaluating the effectiveness of possible interventions.

The lack of follow-up data, due to the patient’s voluntary discharge, prevents a thorough understanding of the natural course of the vertebrobasilar aneurysm and SS combination. Long-term monitoring could have provided insights into the progression of the condition and potential delayed complications, such as aneurysm rupture.

Although MRA and Doppler ultrasound were used for diagnostic purposes, other advanced imaging techniques such as catheter-based angiography or flow-diversion studies might have provided more comprehensive information on the hemodynamic alterations and aneurysm characteristics.

The study lacks a comparative analysis with other cases of vertebrobasilar aneurysms or SSS, which could provide a broader context and enhance understanding of potential variations in presentation and treatment response.

Future directions

The rarity and complexity of Wallenberg syndrome secondary to vertebrobasilar aneurysms associated with SSS present unique challenges for vascular neurosurgery. Future advancements in this field can be driven by innovations in diagnostic and therapeutic approaches, including the integration of 3D printing, augmented reality (AR), and advanced imaging techniques.

Application of Three-Dimensional Printing for Presurgical Planning

3D printing technology has shown the potential to enhance preoperative planning for complex vascular cases. For vertebrobasilar aneurysms associated with SSS, 3D models based on imaging data (CT, MRI, or angiography) allow surgeons to examine the patient’s unique anatomy [31-33]. These models provide a tangible representation of the aneurysm’s location and relationship with critical structures, such as the brainstem and cranial nerves, assisting in selecting appropriate techniques [33-35]. Surgeons can simulate different strategies, assessing potential risks before surgery [35-37]. 3D printing can create custom devices, such as stents, tailored to the patient’s anatomy [37,38].

Augmented Reality in Neurosurgical Procedures

AR offers potential benefits in complex vascular surgeries by overlaying digital images in the surgical field. In deep areas such as the vertebrobasilar junction, AR provides real-time imaging overlays, aiding surgeons in visualizing critical blood vessels and their relation to the aneurysm without surgical exposure [39,40]. Integrated with surgical navigation systems, AR updates the aneurysm’s size, position, and flow dynamics during the procedure, reducing the risk of complications [41]. AR enables surgeons in training to practice procedures in rare cases in a virtual environment before real surgery [42].

Use of Advanced Imaging Technologies

More advanced imaging technologies, such as four-dimensional flow MRI, could provide real-time, dynamic visualization of blood flow in cases involving SSS. This would improve the understanding of retrograde flow and its role in aneurysm formation, aiding in therapeutic decision-making [43-45].

Establishing a clinical registry or multi-institutional studies could advance the understanding and management of vertebrobasilar aneurysms associated with SSS, providing data on outcomes, procedural successes, and complications [43]. Collaboration between centers would help identify the best treatment protocols and evaluate emerging technologies in surgical practice.

## Conclusions

This case of Wallenberg syndrome secondary to a vertebrobasilar aneurysm associated with SSS highlights the complex interplay between vascular pathologies and neurological presentations. The rarity of both conditions, compounded by the hemodynamic alterations from SSS, adds a unique dimension to the clinical challenge of diagnosing and managing vertebrobasilar aneurysms. In this patient, the retrograde flow caused by subclavian artery stenosis likely contributed to increased hemodynamic stress at the vertebrobasilar junction, fostering aneurysm development. The clinical presentation, characterized by the classic features of Wallenberg syndrome, underscores the importance of considering vascular anomalies as potential underlying causes in similar cases.

This report also emphasizes the critical role of advanced imaging techniques such as MRA and Doppler ultrasound in diagnosing and understanding the hemodynamic changes that contribute to such rare vascular anomalies. Although the patient declined invasive therapeutic interventions, which limited the scope of possible treatment options, this case reinforces the importance of a multidisciplinary approach to managing vertebrobasilar aneurysms, particularly when complicated by conditions such as SSS.
